# Supplementary material for: Estimating the current burden of Chagas disease in Mexico: A systematic review and meta-analysis of epidemiological surveys from 2006 to 2017
Source: PLoS Negl Trop Dis. 2019 Apr 9;13(4):e0006859. doi: 10.1371/journal.pntd.0006859 (PMC6474657; doi:10.1371/journal.pntd.0006859)
Supplement: S1 Flow Diagram — (PDF) [file pntd.0006859.s002.pdf]

PRISMA 2009 Flow Diagram

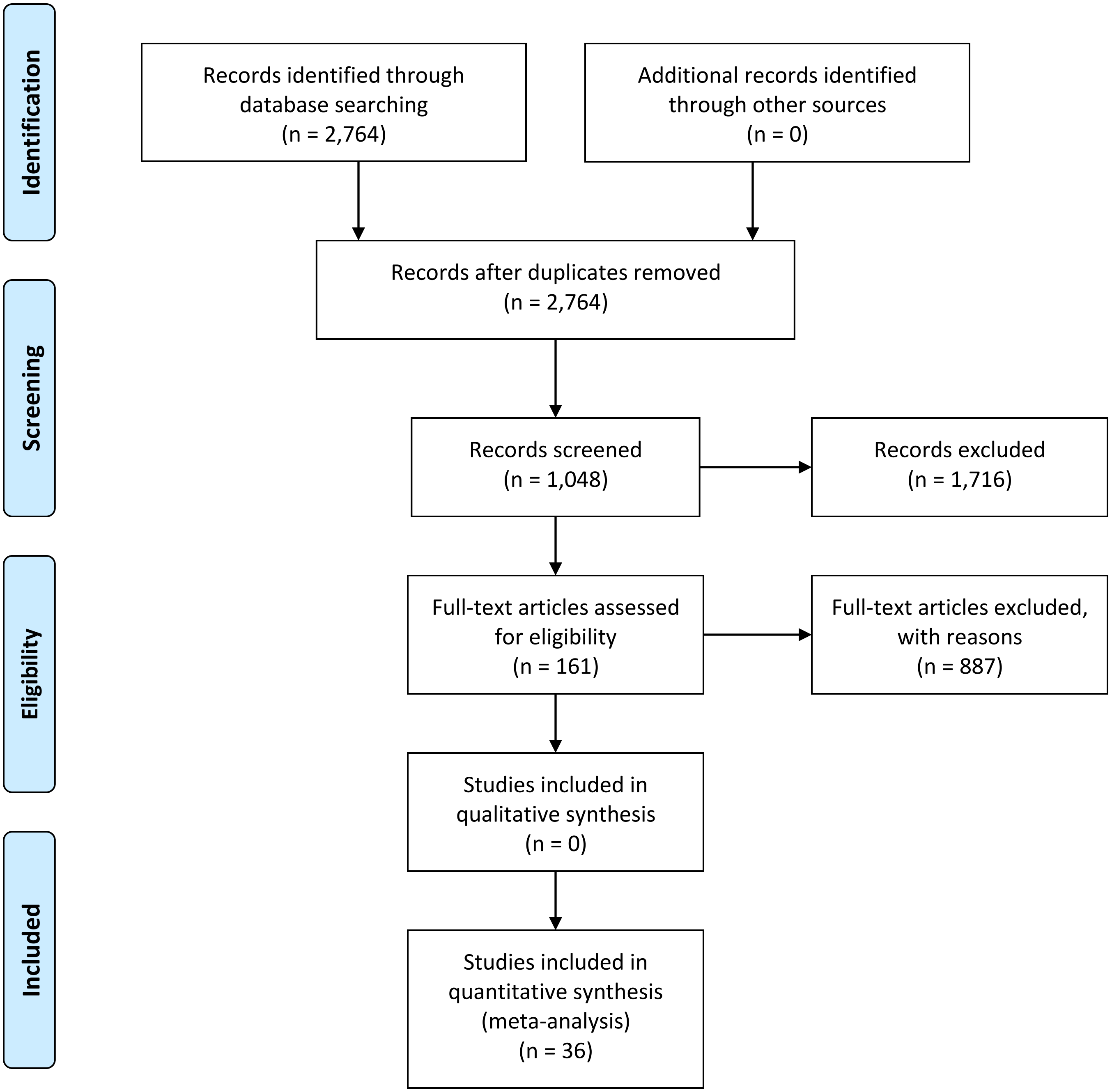

From: Moher D, Liberati A, Tetzlaff J, Altman DG, The PRISMA Group (2009). *P*referred *R*eporting *I*tems for *S*ystematic Reviews and *M*eta-*A*nalyses: The PRISMA Statement. PLoS Med 6(7): e1000097. doi:10.1371/journal.pmed1000097

For more information, visit www.prisma-statement.org.
